# Design and use of chimeric peptides in a new non‐destructive ecological process applied to the extraction of all trans/9‐cis β‐carotene isomers from *Dunaliella salina*

**DOI:** 10.1002/fsn3.2809

**Published:** 2022-03-14

**Authors:** Soumaya Kouidhi, Wissem Mnif, Nada Alqarni, Soukaina Abdelwahed, Alaeddine Redissi, Nihel Ammous, Boulbaba Selmi, Ali Gargouri, Sami Achour, Ameur Cherif, Amor Mosbah

**Affiliations:** ^1^ Laboratory (BVBGR)‐LR11ES31 University Manouba ISBST Biotechnopole Sidi Thabet Ariana Tunisia; ^2^ 488770 Department of Chemistry Faculty of Sciences and Arts in Balgarn University of Bisha Bisha Saudi Arabia; ^3^ Laboratory of Biotechnology and Valorisation of Bio‐GeoRessources Higher Institute of Biotechnology of Sidi Thabet BiotechPole of Sidi Thabet University of Manouba Ariana Tunisia; ^4^ Laboratory of Molecular Biotechnology of Eukaryotes Center of Biotechnology of Sfax University of Sfax Sfax Tunisia; ^5^ Laboratory of Bioresources Integrative Biology and Valorization Higher Institute of Biotechnology of Monastir University of Monastir Monastir Tunisia

**Keywords:** chimeric peptides, *Dunaliella salina*, ecological extraction, industrial scale, *β*‐carotene

## Abstract

Recently, β‐carotene has gained tremendous importance as a bioactive molecule due to the growing awareness of the harmful effects of synthetic products. β‐carotene is a high‐value natural pigment that has the highest demand in the global carotenoid market owing to its proven antioxidant properties relevant for several diseases. To date, *Dunaliella salina* is the most important producer of natural β‐carotene and is the subject of important industrial efforts. However, the extraction of β‐carotene remains challenging since all the proposed techniques present a risk of product contamination or loss of quality due to solvent residuals and low yields. The purpose of this study was to set up a green, ecological, and innovative process of extraction of the two major β‐carotene isomers from the halophilic microalgae *Dunaliella salina*. Based on molecular modeling, docking, and drug design, we conceived and synthesized two chimeric peptides (PP2, PP3) targeting specifically the two major isomers: all‐trans or 9‐cis β‐carotene. The experimental protocol used in this study demonstrated the ability and the efficacy of those two peptides to cross the cell membrane and bind with high affinity to β‐carotene isomers and exclude them toward the extracellular medium while preserving the integrity of living cells. Interestingly, the tested peptides (PP2, PP3) exhibit significant β‐carotene extraction yields 58% and 34%, respectively, from the total of the β‐carotene in microalgae cells. In addition to its simplicity, this process is fast, independent of the source of the β‐carotene, and selective. These results would allow us to set up a green, ecological, and very profitable process of extraction from microalgae containing high amounts of β‐carotene. Our innovative approach is highly promising for the extraction of *Dunaliella salina* biomass on an industrial scale.

## Graphical abstract

β‐Carotene is a high‐value natural pigment that has the highest demand in the global carotenoid market owing to its proven antioxidant properties relevant for several diseases. However, the extraction of β‐carotene remains challenging since all the proposed techniques present a risk of product contamination or loss of quality due to solvent residuals and a low yield. The purpose of this study was to set up a green, ecological, and innovative process of extraction of the two major β‐carotene isomers from the halophilic microalga *Dunaliella salina*.

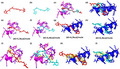

## 1 INTRODUCTION

Carotenoids are natural pigments that are responsible for the bright colors of many fruits, vegetables, and algae. They also might be found in photosynthetic organisms like fungi and bacteria (Maoka, 2020). Most of these carotenoids have been extensively studied for their health benefits as antioxidant or anti‐cancer agents. Moreover, they are widely used as valuable natural food colorants to the food industry. This is argued by the fact that carotenoids could not be synthesized by human beings from endogenous precursors; they are mostly acquired from the diet. Thereby, the global market for all carotenoids was US$ 1.5 billion in 2017 and is expected to reach US$ 2.0 billion by year 2026 (Ambati et al., 2019).

β‐Carotene is one of the extensively used carotenoids, mainly in food, drug, and cosmetic industries (Krinsky, 2000). β‐Carotene is a lipophilic high‐value compound and a precursor of vitamin A (retinol) that belongs to the chemical class of isoprenoids (terpenoids) and is indispensable to allow proper function of the retina, epidermis, and mucous membranes (Chichili et al., 2005). Several studies showed antioxidant properties of β‐carotene in vitro and in animal models. Thus, β‐carotene has gained interest especially for its nutraceutical properties and its ability to prevent several diseases such as cardiovascular disease, cancer, inflammation, diabetes, and neural problems, including the possible prevention and treatment (Mayne, 1996; Nasri et al., 2014; Stahl & Sies, 2005). Recently, researchers found that β‐carotene supplementation shows promising improvements in microbiota composition, and a reduced risk of diseases caused by microbiota dysbiosis (Lyu et al., 2018).

Nevertheless, β‐carotene is composed of two major stable geometric isomers: all‐trans β‐carotene, the common natural form of this pigment, and 9‐cis β‐carotene. Several experimental studies have suggested that the 9‐cis β‐carotene has a higher antioxidant and anti‐cancer potency than that of the all‐trans isomers (Levin & Mokady, 1994; Xuebo & Osawa, 2007). 9‐cis β‐carotene is one of the most potent precursors of retinoids. Recent evidence has suggested that 9‐cis β‐carotene might serve as an effective treatment for a range of retinoid dystrophies, including type 2 diabetes, obesity, certain types of cancer (breast, cervical, ovarian, and colorectal), mild, chronic plaque psoriasis, and a range of cardiovascular diseases (Hieber et al., 2000). To date, the large‐scale β‐carotene production is performed by either chemical synthesis using or from limited selective natural resources. However, the synthesis of 9‐cis β‐carotene is scarce and difficult compared to all‐trans forms. The difficulty in such synthesis is reflected by the high price of 9‐cis β‐carotene reaching €500,000/g (Harvey & Ben‐Amotz, 2020).

*Dunaliella Salina* is a genus of unicellular algae belonging to the family Polyblepharidaceae. In stress conditions (high salinity, high light intensity, or nutrient starvation), *Dunaliella Salina* produces naturally high amounts of carotenoids (up to 14% of its dry mass) as a protection mechanism. This massive accumulation of β‐carotene has led to interesting biotechnological applications (Tafreshi & Shariati, 2009; Xu & Harvey, 2019). In fact, these microalgae are commercially advantageous to cultivate for industrial applications as they exhibit fast growth with simple nutritional requirements. Interestingly, *Dunaliella Salina* naturally produce large concentrations of all‐trans (50% of β‐carotene composition) and 9‐cis β‐carotene (>40% of β‐carotene composition). With this isomer composition, the algae‐derived pigment is claimed to be healthier than the synthetic products (Harvey & Ben‐Amotz, 2020; Xu et al., 2018).

Previous studies have reported several optimum processes for β‐carotene extraction from *Dunaliella salina*. Mainly, the described methods used edible oil, with or without conventional organic solvent, where ethanol and acetone were identified as the best co‐solvent for extraction of β‐carotene from *D. salina*. Furthermore, liquid or supercritic CO_2_ extraction crystallization, ultrasonic‐assisted extraction, and microwave‐assisted extraction were also highlighted (Hejazi et al., 2002; Poojary et al., 2016).

All these methods require the destruction of large amounts of biological biomass to obtain the pure molecule. In addition, the risk of product contamination or loss of quality due to organic solvent residuals in the extracts provokes a rising need for innovative extraction methods. One potential approach in this direction is a new non‐destructive process based on peptide for β‐carotene extraction from *D. salina*. In fact, the importance of this process lies in its advantages over cited methods. This new process is based on the use of chimeric peptides formed by a cell‐penetrating peptide fused with β‐carotene‐binding peptides targeting all‐trans or 9‐cis β‐carotene, in order to obtain a high yield and pure molecules without destroying the microalgae cells.

## 2 MATERIAL AND METHODS

### 2.1 Conception, molecular model, and docking of the peptides

The models of the penetrating peptide, the specific peptide targeting β‐carotene, as well as the chimeric peptides were constructed using the PEP‐FOLD3 software (Lamiable et al., 2016). Then refined using the NAMD software (Humphrey et al., 1996; Wong & Goscinski, 2012). Before the minimization with NAMD, the peptide structure files are generated with VMD software (Humphrey et al., 1996; Wong & Goscinski, 2012). During the minimization cycle with NAMD, 1000 minimization cycles were used for all models at a temperature of 25°C. Minimization was performed under the Charm force field.

The structure of cis and trans β‐carotene was downloaded from the PubChem database and minimized with the NAMD software. The chimeric peptides and minimized cis and trans β‐carotene were used as partners in the docking using Autodock Vina software.

During the docking procedure, all chimeric peptides were used as receptor, almost of his bonds were defined as no rotatable and cis or trans β‐carotene were used as a ligand and kept rigid. Grid map representing the target was constructed with the dimensions of the peptides.

The free three‐dimensional (3D) molecular structures of the receptor or complexed with cis or trans β‐carotene were visualized using the molecular visualization software PyMOL (http://www.pymol.org/) (Brunger et al., 2009) and the ligand–peptide complex interaction obtained was visualized by the Python Molecular viewer 1.5.6 (Sanner, 2005) and the two‐dimensional representation was performed by Discovery Studio Visualizer 20.1.0 (Biovia, 2020).

### 2.2 Solid‐phase synthesis of selected peptides

Solid‐phase synthesis was manually performed using amino acids with Fmoc amino protected function. The resin used is a Rink amide resin, it has a molecular weight of 495.575 g/mol, and its extent of labeling is 0.5 mmoles/g. In each experiment, 0.125 mmole of a rink amide resin was used as a starter. The amino acid coupling step requires an alkaline medium, provided by the addition of DIPEA, as well as catalysts such as Pybop or HcTU or EDC. The elimination of the Fmoc‐protecting group is generally ensured by a strong base such as piperidine. For the amino acid side chain, the elimination of their protective groups as well as solid polymers is conducted using a strong acid, such as TFA. To get rid of amino acid excess or coupling agents, simple washing with DMF is necessary (Mosbah et al., 2019). To ensure that the amino acid coupling is done, the “Kaiser test” was performed. This test requires two solutions A and B whose compositions are as follows: Solution A: a mixture of pyridine (49 ml) + 20 ml ethanol +0.8 g phenol +1 ml of KCN (1 mM); and Solution B: a mixture of 1 g ninhydrin +20 ml ethanol.

### 2.3 Synthesized peptides verification

After achieving the synthesis of desired peptides: Seq 1: VAGWWWWGTRRMKWKK‐NH_2_, and Seq 2: YGYVPSRWWGTGRRMKWKK‐NH_2_, they were cleaved from the solid phase using a strong acid: trifluoroacetic acid (TFA), 0.5% water, and 0.5% triisopropylsilane (TIS) as scavengers. Peptides precipitation requires treatment with cold ether (2v/v) followed by centrifugation. The resulting white pellet proves the presence of a synthesized peptide. We obtained, after lyophilization, unpurified peptides in spongy form, which were weighed to determine the quantities of each one and calculate synthesis yield. Theoretical molecular weights of peptides were calculated using Protein Prospector software and the experimental mass of each peptide was verified by LCMS.

### 2.4 Experimental activity assay of β‐carotene extraction from *Dunaliella salina* via peptide system

#### 2.4.1 Microalgae culture

Culture conditions of *Dunaliella salina* were settled according to scientific research for a maximum production of β‐carotene (culture medium composition, culture temperature, UV intensity, salinity, etc.) (Zarandi‐Miandoab et al., 2019). For optimum culture, the temperature was maintained at 21°C, a light intensity of 18,000 lux was applied, the salinity was 43 gr/L, and the nitrogen concentration was 45 mg/L. Cultures were carried out in 2 L glass flasks and maintained in controlled conditions until pinkish color appeared. Microalgae cells were counted per milliliter of culture using a Malassez cell so that the peptide concentration needed to extract β‐carotene can be estimated.

After a sonication, 5 ml of a culture was centrifuged for 30 min at 5000 rpm in centricons (Millipore, ultracel 10 kDa). The concentrated filtrate obtained is subjected to an OD reading at 450 nm to estimate the initial β‐carotene concentration. An OD of standard range of β‐carotene was previously realized.

One‐hundred microliters of the two peptide solutions, PP2 and PP3, prepared at 250 mg/ml in water, pH 7, was added to 5 ml of the microalgae solutions at 10^6^cell/L to extract β‐carotene. A reading of the OD at 450 nm was carried out after a different reaction time of 30 min, 1 h, and 1 h 30 min between the peptide and the microalgae and a centrifugation of 30 min at 5000 rpm in centricons (Millipore, ultracel 10 kDa). The blank used in this experiment was undergoing the same procedure without the peptide addition. All the manipulations described here were performed in triplicates.

#### 2.4.2 *Dunaliella salina* culture after β‐carotene extraction

To ensure the validity of the strategy adopted in the extraction of β‐carotene from microalgae, a control step is performed to verify cell viability and normal multiplication. In fact, microalgae solutions which have been treated with peptide solutions were cultivated under optimal conditions of salinity and luminosity after having noted their OD at 750 nm (corresponding to chlorophyll).

A microscopic observation (×100) of the *Dunaliella salina* culture was carried out to verify the cell growth state after β‐carotene extraction.

## 3 RESULTS

### 3.1 Conception of the chimeric penetrating and β‐carotene‐binding peptides

In this step, we firstly selected peptide sequences targeting β‐carotene from the literature (Binder et al., 2016). All peptides were described as specifically binding to β‐carotene, but the authors did not specify if they are belonging to cis or trans β‐carotene. To determine their specificity toward the two above isomers, we carried out a 3D model of each peptide.

**FIGURE 1:**
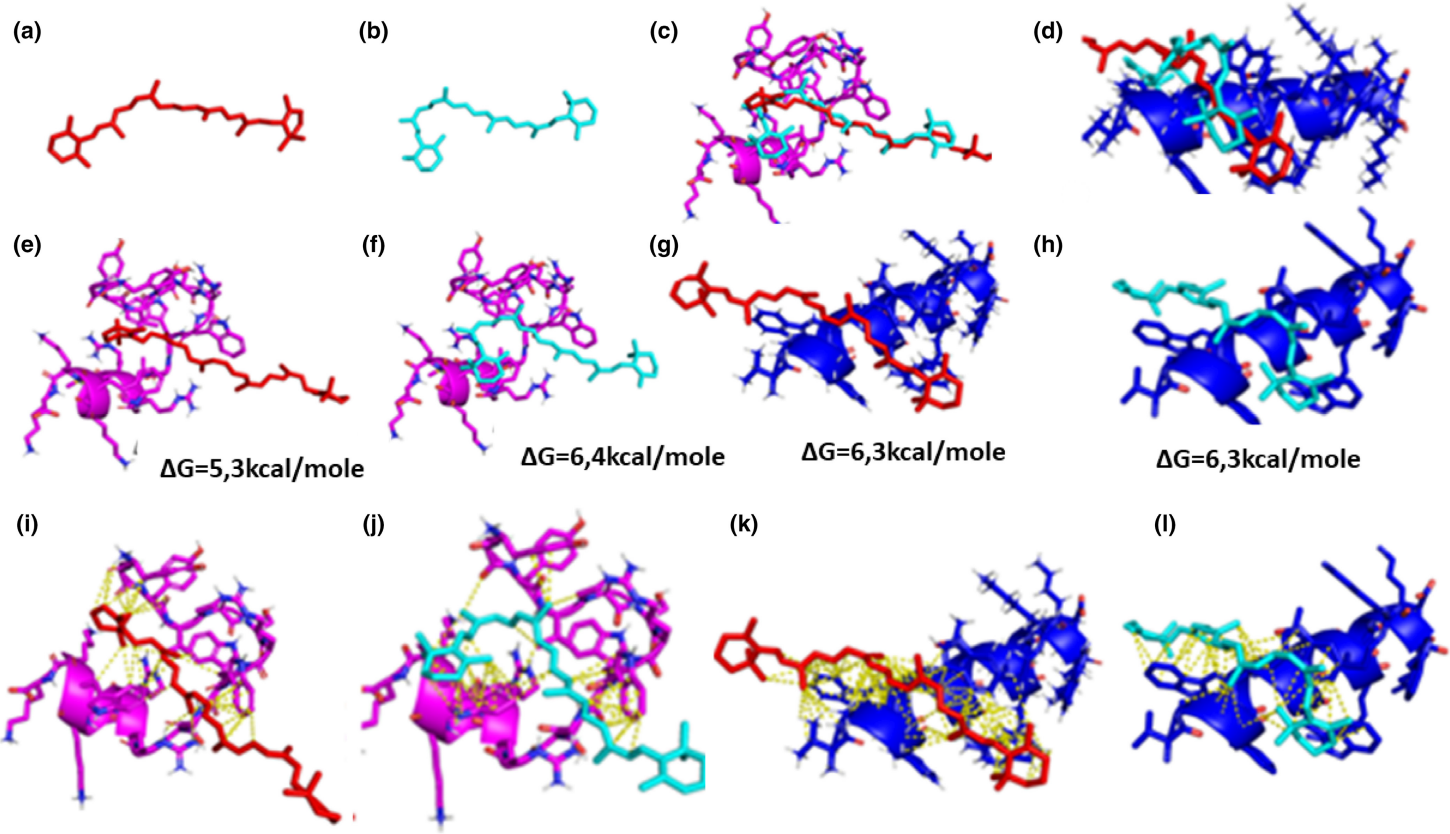
Molecular Modulization, docking, and interaction of PP2 and PP3 with cis–trans conformations of β‐carotene. (a) Structure of trans β‐carotene (sticks), (b) structure of 9‐cis β‐carotene (sticks), (c) superimposition of the result of docking (Autodockvina) of the trans β‐carotene (red) and 9‐cis β‐carotene (cyan) to the chimeric peptide PP3. (d) Superimposition of the result of docking (Autodockvina) of the trans β‐carotene (red) and 9‐cis β‐carotene (cyan) to the chimeric peptide PP2. (e) Docking result of the most energetically favorable solution of the complex PP3/trans β‐carotene, the energy of interaction of the most favorable solution was printed. (f) Docking result of the most energetically favorable solution of the complex PP3/cis β‐carotene, the energy of interaction of the most favorable solution was printed. (g) Docking result of the most energetically favorable solution of the complex PP2/trans β‐carotene, the energy of interaction of the most favorable solution was printed. (h) Docking result of the most energetically favorable solution of the complex PP2/cis β‐carotene, the energy of interaction of the most favorable solution was printed. (i) The complex PP3/trans β‐carotene was stabilized by a few of hydrophobic interaction. (j)The complex PP3/cis β‐carotene was stabilized by many hydrophobic interaction. (k): The complex PP2/trans β‐carotene was stabilized by a many hydrophobic interaction. (l) The complex PP2/cis β‐carotene was stabilized by many hydrophobic interactions

Two specific peptides (P1: VAGWWWWGT and P2: YGYVPSR) (Figure 1a) were selected from the phage display library described by Binder and his collaborators in 2013 and Janssen and his collaborators in 2004 (Binder et al., 2016). Peptide's selection was based on peptide ability to bind trans β‐carotene isomer and cis β‐carotene isomers. Using docking binding energy, we select one peptide (P1) with a high affinity to the trans isoform of β‐carotene and a peptide (P2) with a high affinity to the cis form.

After that, we used a penetrating peptide, whose sequence is as follows: RRMKWKK (Figure 1a) from the cell‐penetrating database (Gautam et al., 2012; Watson et al., 2017). In fact, this peptide is dedicated to cross the cell membrane and vesicle.

The chimeric peptides (PP2 and PP3) were then conceived and verified by molecular modeling using PEP‐FOLD3 software (Lamiable et al., 2016), minimization by NAMD software (Figure 1), and AutoDock Vina (Trott & Olson, 2010) for the docking to the cis and trans β‐carotene (Figure 1). The structure of all models was validated by Procheck (Laskowski et al., 1993).

In fact, the two β‐carotene‐binding peptides were attached to a penetrating peptide for a dual role: penetrating the cell membrane and extracting cis and/or trans β‐carotene molecules from *Dunaliella salina* cells. We assume that peptides should be able to enter and exit cells by the same mechanism.

The model was validated by docking the two chimeric peptides to the cis and trans β‐carotene models and an adjustment was made in order to increase the interaction of the fixing or binding peptide to cis and trans β‐carotene (Figure 1). The binding energy of the peptides PP2 was the same for the trans and cis β‐carotene with an energy of binding near to ΔG = −6.3 Kcal/mole. In contrast, the energy of binding of the PP3 was dramatically different between the cis and trans β‐carotene with ΔG= −6.4 Kcal/mole corresponding to the cis isomer and ΔG= −5.2 Kcal/mole for the trans β‐carotene. This result suggests a high selectivity of the PP3 toward the cis β‐carotene form.

### 3.2 Synthesis of the chimeric penetrating peptides β‐carotene‐binding peptides

Peptides were then synthesized by solid‐phase peptide synthesis. Indeed, 0.125 mmol of Fmoc amide resin is used for each peptide to obtain 222 mg and 235 mg, respectively, of peptidyl resin, giving after cleavage 77 mg of PP2 peptide and 89 mg of PP3. The results of peptides synthesis were shown in Tables 1 and 2. The mass of those peptides was verified using LC‐MS approach; Crude peptides were used in β‐carotene extraction.

**TABLE 1:** Yield of PP2 and PP3 Synthesis by SPPS

	PP2	PP3
Estimated theoretical molar mass (g/mol)	2219.68	2442.91
Experimental molar mass obtained (g/mol)	2220.12	2443.16
Experimental mass obtained (mg)	222	235
Number of active resin sites (mmol)	0.125	
Expected theoretical mass expected (mg for 0.125 mmol)	277.46	305.36
Estimated theoretical molar mass (g/mol) 222/277.36	80	76.96

**TABLE 2:** Estimation of attached peptide molecules per microalgae cell

	PP2	PP3
Mass of stock solution (mg/mmol)	77	89
Mass used (mg/mmol)	25	25
Number of cells per 5 ml	5000	

### 3.3 β‐Carotene extraction from Dunaliella salina via peptide system

**FIGURE 2:**
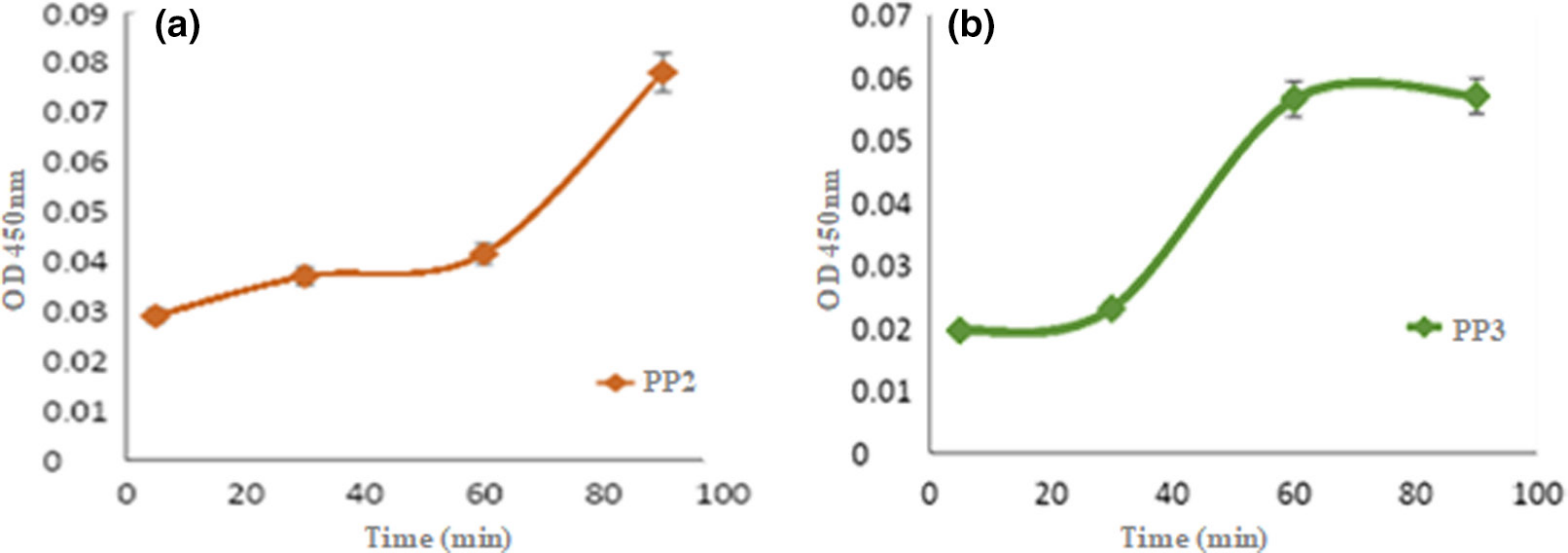
Kinetic of β‐carotene extraction using the chimeric peptides (a) PP2 and (b) PP3

The synthesized peptides were then tested for their ability to perform extraction of β‐carotene from *Dunaliella salina*. The evolution profile of β‐carotene extraction by peptide PP2 and PP3 is shown in Figure 2. It can be clearly seen that the increase in extraction time significantly enhances the β‐carotene yields. The two curves showed that the extraction of β‐carotene increases with time, which indicates that PP2 and PP3 allow both dose‐dependent extraction of β‐carotene. This is expressed by an increase in OD at 450 nm until reaching a value of 0.078 for PP2, which corresponds to 58% of the total content of β‐carotene in the solution and a value of 0.057 for PP3 corresponding to 38% of the total content of β‐carotene in the solution. Also, visually the middle becomes pinkish. The quantity of β‐carotene extracted at a fixed dose confirmed by the projection of the standard range curve shows that PP2 allows better extraction compared to PP3. Based on a statistical analysis of data, it was found that a maximum extraction efficiency of β‐carotene could be achieved using PP2 at 90 min, but PP3 reaches the maximum of carotenoid extraction at 60 min before PP2 (at 30 min).

### 3.4 Microalgae culture after β‐carotene extraction

After β‐carotene extraction, a culture of the remaining microalgae was performed for 7 days and the OD was measured on the last day to ensure growth recovery and multiplication.

**FIGURE 3:**
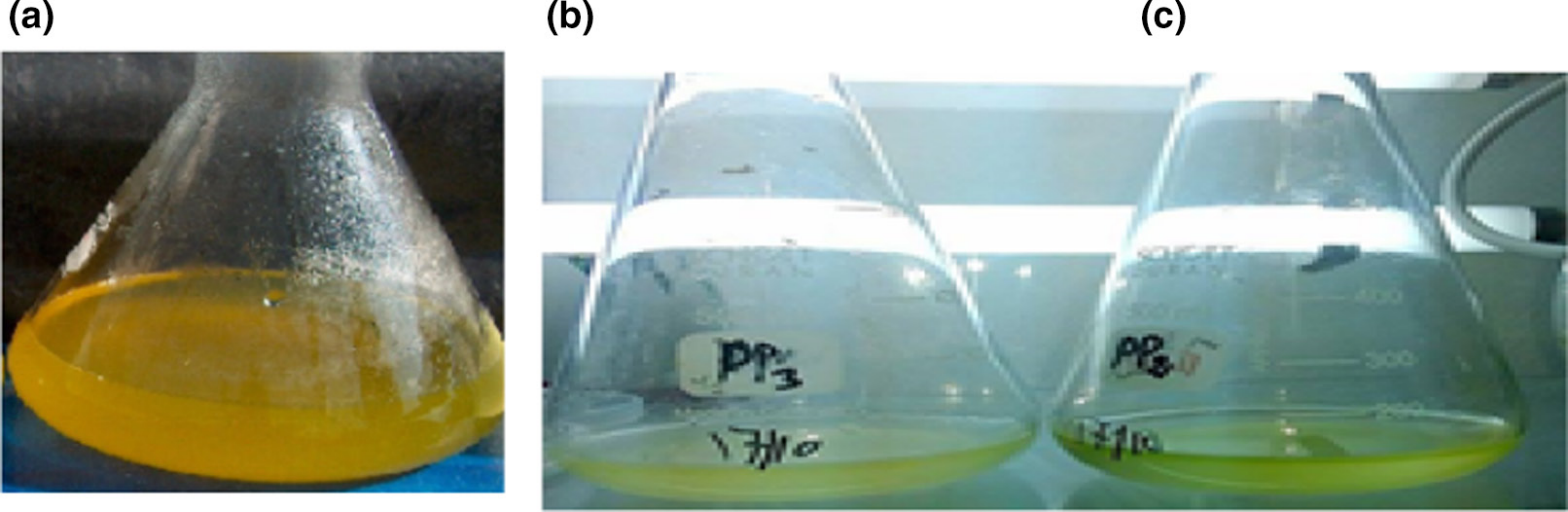
Cultivation of *Dunaliella salina* under optimal conditions before and after β‐ carotene extraction. (a) Immediately before extraction. (b) After extraction using PP3. (c) After extraction using PP2

**FIGURE 4:**
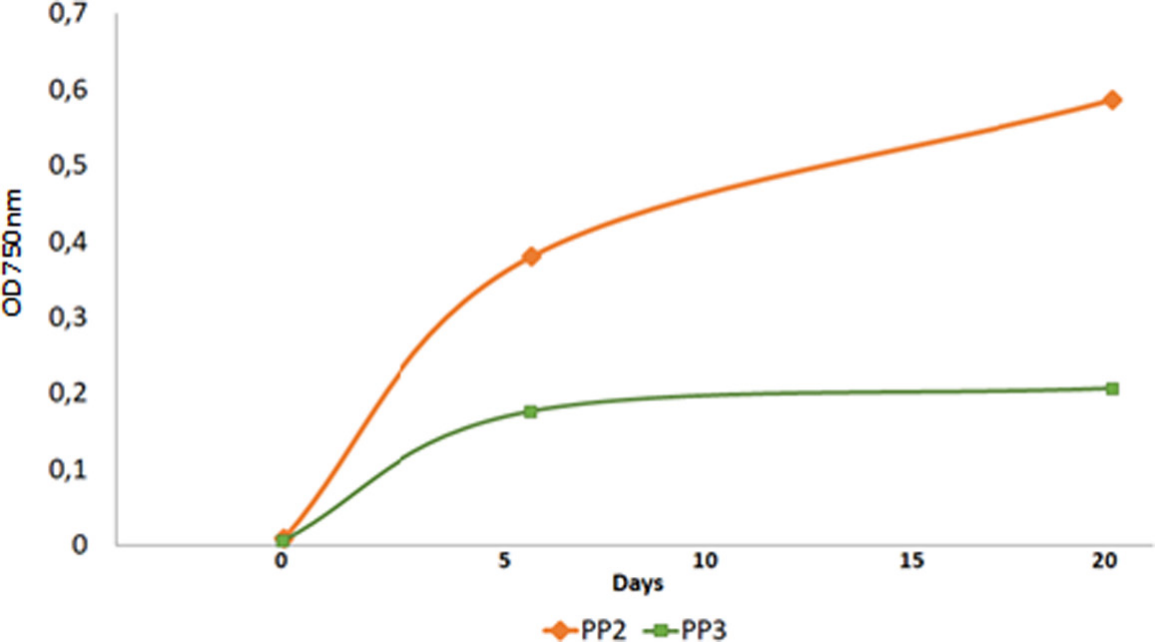
Growth evaluation of *Dunaliella salina* cells after β‐carotene extraction by PP2 and PP3

**FIGURE 5:**
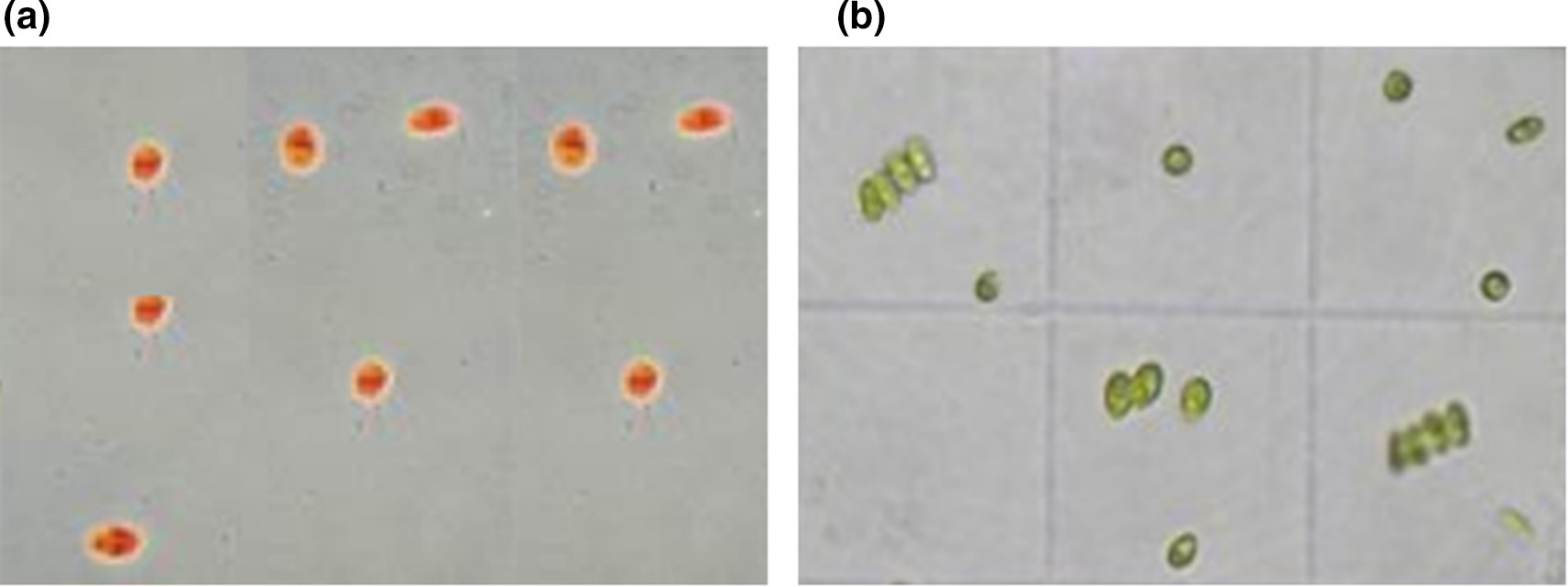
Microscopic observation of *Dunaliella Salina before* (a) and after (b) β‐carotene extraction (×100)

The absorbance at 750 nm of the *Dunaliella* culture has increased in comparison to the initial culture. This increase in OD was reflected in the color shift from light pink to dark green of the microalgae culture, which proves that cells are in a good state of growth by producing chlorophyll pigments (Figure 3). In fact, the evaluation of cell growth after β‐carotene extraction by PP2 and PP3 is shown in Figure 4, indicating a strictly increasing curve in accordance with time evolution. Our results suggest that following specific secondary metabolites peptides‐based extraction, there was no cell lysis. These results are confirmed by a microscopic observation (x100) of the *Dunaliella* culture which shows that the cells are in a state of growth and they are mobile (Figure 5).

## 4 DISCUSSION

At present, the carotenoids market is projected to grow to USD 2.0 billion by 2026. In the field of biotechnology, there is a big attention toward natural carotenoids especially to β‐carotene production. Hence, the β‐carotene extraction from natural resources is experiencing an exponential evolution in several fields of industrial production. In fact, it has a highly added value in preventing many diseases, in cosmetics and as natural colorant in food (Bogacz‐Radomska & Harasym, 2018; Wichuk et al., 2014). In addition, some forms of β‐carotene, like the 9‐cis β‐carotene, are difficult to synthesize chemically and overpriced (estimated €500,000/g). Among the natural sources, *Dunaliella salina* is a green microalgae that offers the highest concentration of natural β‐carotene (Xu et al., 2018). Despite the huge efforts made during the last decade to develop optimum processes and methods, extraction of natural β‐carotene from *Dunaliella salina* remains challenging because of several limitations such as final product contamination, low yields or high costs, and degradation of the biomass (Khan et al., 2018).

In the current study, we aimed to set up an innovative strategy that allows at the same time a high yield product and a sustainable process for the industrial development of algae. Thus, we performed for the first time the extraction of β‐carotene from *Dunaliella salina* using specific peptides targeting β‐carotene (both trans and cis forms) denoted with cargo peptide. Cargo peptides or cell‐penetrating peptides are known in the literature for their roles in the transport of even large drug molecules. The mechanisms used by these peptides to cross cell membranes are well described and range from direct passage to specific port formation or to vesicle formation followed by endocytosis or exocytosis (Böhmová et al., 2018; Falanga et al., 2020; Gestin et al., 2017; Kardani et al., 2019; Ramsey & Flynn, 2015; Sahni et al., 2020). The passage of these peptides through the cell membrane is very rapid (5–20 min) (Zorko & Langel, 2005). These penetrating peptides have the capacity to cross the cell membrane in both directions as already described in the literature (Popa et al., 2019).

Interestingly, our pilot‐scale results showed a significant yield of final extracted β‐carotene. According to our docking models, the two chimeric peptides do not show the same energy of interaction toward cis and trans β‐carotene. The chimeric PP2 seems to interact equally to both isoforms. However, the chimeric PP3 seems to be more specific to the cis β‐carotene form. Consequently, the yield of extracted β‐carotene using PP2 was higher than using PP3. Thereby, these differences suggest that PP2 may be able to extract both trans and cis β‐carotene fraction, whereas PP3 is more specific to target only the cis β‐carotene fraction. In addition, the obtained results using this pioneer method are in line with those reported previously in the *Dunaliella salina* model. In fact, it is well described that *Dunaliella salina* accumulates up to 50% of all trans β‐carotene and up to 40% of 9‐cis β‐carotene (Harvey & Ben‐Amotz, 2020).

Traditionally, the most performed methods for β‐carotene extraction used organic solvent extraction, which may be toxic and has some pollution concerns (Adadi et al., 2018). As an alternative, the use of supercritical CO2 (scCO2) has been developed to overcome some limitations of traditional extractions. Although it has better stability than the organic solvent extraction, the use of scCO2 needs a dried biomass, which is energy intensive (Patel et al., 2019).

It is clear that β‐carotene extraction with a peptide system seems to be different from other described methods (Poojary et al., 2016). In fact, our extraction system could be directed according to the extraction target as it is possible to design specific peptide sequences to extract specific molecules such as chlorophyll and phycobiliproteins.

This process is extremely accurate since it is based on binding molecules in accordance with a specific fixing pattern, allowing a specific extraction at almost 100%. While other extraction techniques, like supercritical coupled with adsorbents (silica gel), allow the extraction of β‐carotene isomers, as described by Mendes and his collaborators in 2003 (Mendes et al., 2003; Srinivas & King, 2010), this process could be qualified as the most specific extraction technique as it extracts pure β‐carotene molecules which could be intended for pharmaceutical or cosmetic use. Nevertheless, this pioneer method could overcome the highly expensive price of the chemical synthesis of the cis β‐carotene form.

Nevertheless, our method allowed the conservation of biomass that could be reused for further extractions. From an environmental perspective, the described process is non‐destructive and eco‐friendly. Indeed, the extraction of β‐carotene from microalgae compared to conventional methods, such as supercritical carbon dioxide and organic solvent extraction (Riyahi & Shimeld, 2007), offers several advantages such as cheaper and easier extraction, higher yields, and no seasonal variations (in comparison to plant source).

However, long‐term exploitation of algal resources entails a lack of raw materials that consequently causes ecological imbalance and serious damages. The described process is considered as a “Green Process” thanks to its soft characteristic to extract β‐carotene from the algae cell without destroying the cell integrity or functionalities. It is crucial to mention that *Dunaliella salina* and some other algal species have complex life cycles that include, in addition to the division of mobile vegetative cells, the possibility of sexual reproduction (Oren, 2005). The fusion of two gametes of equal size to form a zygote has been documented in several studies. Oren has reported in his review the possibility of two reproduction mechanisms in different salinity conditions. These details about *Dunaliella salina* reproduction limit large‐ scale extraction of β‐carotene by traditional methods. Hence, using the described peptide system to extract β‐carotene proves to be an excellent alternative to preserve cell viability and skirt culture renewal problem.

## 5 CONCLUSION

In the present work, we have performed a new bioprocess of β‐carotene extraction from *Dunaliella salina*, marine microalgae, and a strong producer of this molecule under UV and salinity stress conditions. The described bioprocess is distinguished from other extraction techniques by the ability to obtain β‐carotene produced by *Dunaliella salina* cells without disturbing or affecting the integrity of cell membranes. This is ensured by short‐chain peptides, designed to cross the living cell membrane, to fix the molecule of interest and to bring it to the extracellular environment. This invented bioprocess could be a future alternative for interested industries to produce pure β‐carotene with an eco‐friendly and non‐ destructive process.

## CONFLICT OF INTEREST

The authors declare no conflicts of interest.

## Data Availability

Data available upon request from the last author, Dr. Amor Mosbah (amor.mosbah@isbst.uma.tn).
